# Effects of F-Doping on the Electrochemical Performance of Na_2_Ti_3_O_7_ as an Anode for Sodium-Ion Batteries

**DOI:** 10.3390/ma11112206

**Published:** 2018-11-07

**Authors:** Zehua Chen, Liang Lu, Yu Gao, Qixiang Zhang, Chuanxiang Zhang, Chunwen Sun, Xingying Chen

**Affiliations:** 1 College of Chemistry and Chemical Engineering, Henan Polytechnic University, Jiaozuo 54000, China; chen1861@mail.tsinghua.edu.cn (Z.C.); luliang@binn.cas.cn (L.L.); gaoyu2016@163.com (Y.G.); zhangqixiang@126.com (Q.Z.); 2 School of Materials Science and Engineering, Tsinghua University, Beijing 100084, China; 3 CAS Center for Excellence in Nanoscience, Beijing Institute of Nanoenergy and Nanosystems, Chinese Academy of Sciences, Beijing 100083, China; 4 School of Nanoscience and Technology, University of Chinese Academy of Sciences, Beijing 100049, China; 5 Center on Nanoenergy Research, School of Physical Science and Technology, Guangxi University, Nanning 530004, China; 6 Medical College, Henan Polytechnic University, Jiaozuo 454000, China; chen1861@hpu.edu.cn

**Keywords:** sodium-ion batteries, Na_2_Ti_3_O_7_, F-doping, electronic conductivity

## Abstract

The effects of fluorine (F) doping on the phase, crystal structure, and electrochemical performance of Na_2_Ti_3_O_7_ are studied by X-ray diffraction (XRD), scanning electron microscopy (SEM), and electrochemical measurements. F-doping does not change the crystal structure of NTO, although it has an effect on the morphology of the resultant product. As an anode material for sodium-ion batteries, the specific capacity of Na_2_Ti_3_O_7_ exhibits a 30% increase with F-doping owing to the improved sodium ion diffusion coefficient. F-doped Na_2_Ti_3_O_7_ also displays an enhanced rate capability and favourable cycling performance for more than 800 cycles.

## 1. Introduction

At present, lithium-ion batteries (LIBs) are indispensable for power electronics [1,2]. In the coming decades, LIBs will be used on an increasing scale in the field of utility grid and a future “energy internet” [2]. However, the limited reserves and cost issues of lithium present great challenges for grid application of LIBs. Sodium-ion batteries (SIBs) have recently attracted interest because of their advantages such as low cost, environmentally friendliness, and the availability of rich sodium resources [3,4]. Various SIB electrode materials have been developed recently, including sodium metal phosphate [5], Na_0.44_MnO_2_ [6], Na_0.67_Ni_0.23_Mg_0.1_Mn_0.67_O_2_ [7,8], Na_3_[Ti_2_P_2_O_10_F] [9,10], NaMnFe_2_(PO_4_)_3_ [11], V_2_O_5_ [12], NiCo_2_O_4_ [13], Na_3_V_2_(PO_4_)_3_ [14], Sb_2_O_4_ [15], Na_2_V_6_O_16_ [16], NaFeF_3_ [17], and Na_2_Ti_3_O_7_ [18,19], as well as some hard carbon materials [20,21]. Among these materials, layered Na_2_Ti_3_O_7_ is one of the most ideal choices as negative active materials for SIBs, with favorable electrochemical performance at a relatively low intercalation voltage of approximately 0.3 V as compared to Na^+^/Na and a low activation energy (~0.186 eV) for Na ion insertion [22]. Although the capacity of NTO reaches 200 mAh g^−1^ [23,24], Na_2_Ti_3_O_7_ does not show a better rate capability or longer cycle life due to the large ionic radius of sodium ions (1.02 Å), which makes their diffusion much more difficult [25]. Moreover, the rapid capacity decay during cycling has been ascribed to the decomposition of Na_2_Ti_3_O_7_ in electrolyte [26]. Therefore, improvement of the rate performance of Na_2_Ti_3_O_7_ remains a great challenge [27]. Introducing additional vacancies to accommodate Na^+^ is favorable for reversible and fast ion intercalation and deintercalation. Since fluorine (F) is the most electronegative element, F-doping has demonstrated a certain influence on the crystal structure and stability of Na_x_V_2_O_2_(PO_4_)_2_F, leading to improved electrochemical performance [28].

Here, we studied the effects of F-doping on the electrochemical performance of Na_2_Ti_3_O_7_. The results demonstrated a 30% increase in the specific capacity of Na_2_Ti_3_O_7_ through F-doping thanks to the improved Na^+^ diffusion coefficient. Better rate capability and cycle performance were also observed.

## 2. Preparation and Characterization

### 2.1. The Preparation of Na_2_Ti_3_O_7_ and F-Doping Na_2_Ti_3_O_7_ Samples

All reagents in this study were purchased and used directly. Titanium (IV) oxide, anatase (99.6%) was purchased from Alfa Aesar (Shanghai, China) and anhydrous Na_2_CO_3_ (99.8%) was bought from Sinopharm Chemical Regent Co., Ltd. (Shanghai, China). A solid-phase method was used to prepare Na_2_Ti_3_O_7_. Titanium oxide and sodium carbonate were mixed well at a molar ratio of 1:3. A 5% excess of Na_2_CO_3_ was added to prevent the composition of the Na_2_Ti_16_O_13_ impurity. The mixture was ground for 120 min. Then, the mixture powder was pressed to thin pellets, which were sintered at 800 °C for 10 h in a muffle furnace. After the muffle furnace was cooled to room temperature, the product, Na_2_Ti_3_O_7_ (NTO), was obtained. Na_2_Ti_3_O_7_F_x_ (NTOF_x_) was prepared by the same procedure, except that the calculated amount of NaF was added and the stoichiometric ratio of titanium and sodium was retained. The Na_2_Ti_3_O_7_ samples with different F-doping amounts were labeled as Na_2_Ti_3_O_7_F_x_ (x = 0.1, 0.2, 0.3 and 0.4).

### 2.2. Phase Analysis and Morphology Characterization

The synthesized Na_2_Ti_3_O_7_ and Na_2_Ti_3_O_7_F_x_ samples were characterized by an X-ray diffraction analyzer (PANalytical X’Pert^3^ Powder, Malvern Panalytical, Almelo, The Netherlands) with Cu/Kα (λ = 1.54178 Å) radiation in the 2 theta range from 5° to 80°. The morphologies of NTO and NTOF_0.3_ were measured on a scanning electron microscope (SU8020) (HITACHI, Tokyo, Japan). High-resolution TEM (HRTEM) was tested on an FEI Tecnai, Model G2 F20S-Twin (Brno, Czech), at a working voltage of 200 kV.

### 2.3. Electrochemical Test

For the preparation of the anode electrode, 70 wt% active material (NTO or NTOFs), 20 wt% acetylene black (Alfa Aesar, Shanghai, China), 10 wt% polyvinylidene fluoride (PVDF, KE JING, Hefei, China) as a binder, and *N*-methylpyrrolodone (NMP, Alfa Aesar, Shanghai, China) as a solvent were mixed to form a slurry. Then the slurry was painted on cleaned carbon-coated aluminum foil and dried at 120 °C in a vacuum oven for 10 h. Coin-type cells were used to test the electrochemical performances. All cells were assembled in a glove box filled with argon in which oxygen and water contents were less than 0.1 ppm. The glass fiber 1822-047 membrane (Whatman, Shanghai, China) was used as the separator, 1 M NaClO_4_ in diethyl carbonate (DEC)/dimethyl carbonate (DMC) (1:1 volume ratio) was used as the electrolyte (MJS, Nanjing, China), and metal sodium (Innochem, Beijing, China)was used as the counter electrode. Cyclic voltammetry experiments were carried out with scan rates at a certain range from 0.02 mV s^−1^ to 5 mV s^−1^, with a voltage window between 0.01 V and 2.5 V (versus Na/Na^+^) via CHI 640 E (B15536). Electrochemical impedance spectroscopy (EIS) was tested on an Autolab PGSTAT 302N instrument (Herisau, Switzerland) in a frequency range from 100 KHz to 100 mHz with a bias voltage of 5 mV.

## 3. Results and Discussion

### 3.1. Structural and Composition Characterization

**Figure 1:**
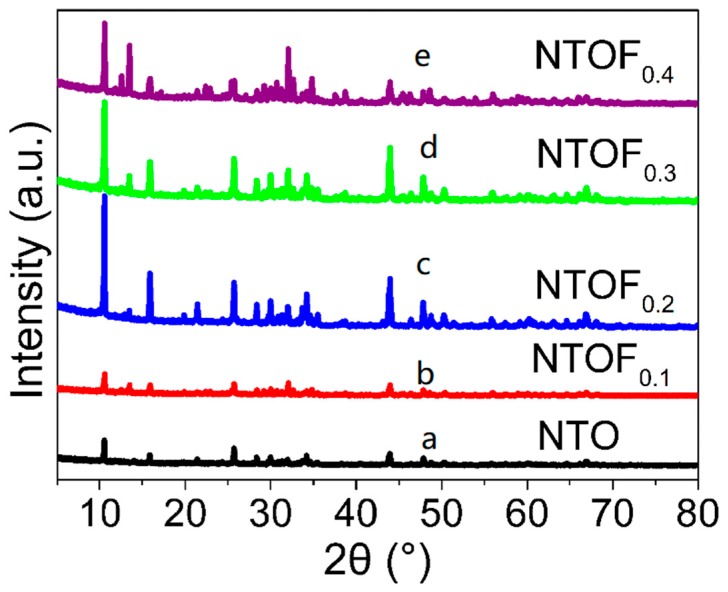
X-ray diffraction (XRD) patterns of NTO and NTOF_x_: (a) NTO; (b) NTOF_0.1_; (c) NTOF_0.2_; (d) NTOF_0.3_; and (e) NTOF_0.4_.

As shown in Figure 1, the Na_2_Ti_3_O_7_ samples with F-doping amounts of 0, 10%, 20%, 30%, and 40% are hereafter referred to as Na_2_Ti_3_O_7_, Na_2_Ti_3_O_7_F_0.1_, Na_2_Ti_3_O_7_F_0.2_, Na_2_Ti_3_O_7_F_0.3_, Na_2_Ti_3_O_7_F_0.1_, and Na_2_Ti_3_O_7_F_0.4_ (x = 0.1, 0.2, 0.3, and 0.4), respectively. As the doping amount of F increases, the intensity of the diffraction peak first increases and then decreases. Several very sharp peaks indicate the high crystallinity of the samples.

**Figure 2:**
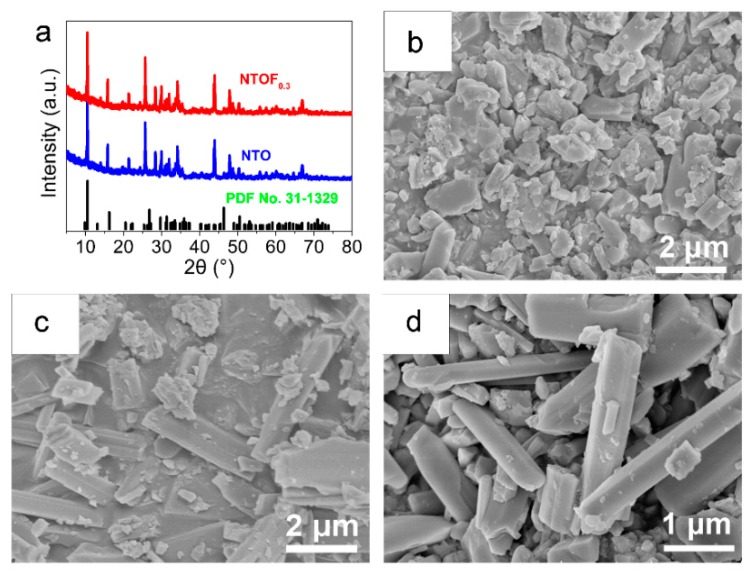
(**a**) XRD patterns of NTO and NTOF_0.3_; (**b**) morphology of NTO; (**c**,**d**) morphology of NTOF_0.3_.

The X-ray diffraction patterns of the NTO and NTOF_0.3_ samples are shown in Figure 2a. All the diffraction peaks correspond to monoclinic Na_2_Ti_3_O_7_ with a (PDF No. 31-1329) P121 space group. No impurity phases were observed, revealing that F-doping does not change the crystal structure of NTO. Figure 2b,c shows the morphology of the prepared NTO and NTOF_0.3_. It is indicated that the size of NTO particles is several micrometres. Most of the NTOF_0.3_ particles show nanorod morphologies (Figure 2c,d), indicating that F-doping has an effect on the morphology of the obtained product.

**Figure 3:**
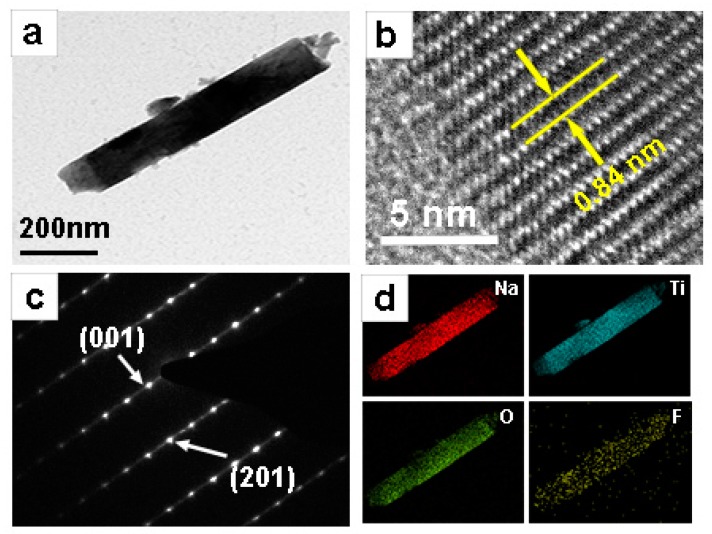
(**a**) TEM image and (**b**) high-resolution TEM (HRTEM) image of the as-prepared NTOF_0.3_; (**c**) selected area electron diffraction (SAED) patterns of NTOF_0.3_; (**d**) the opposite energy-dispersive X-ray spectrometry (EDX) elemental mappings of Na, Ti, O and F.

Figure 3a shows a TEM image of NTOF_0.3_. The diameter of the nanorod is approximately 100 nm, and the length is several micrometres. Figure 3b displays a high-resolution TEM (HRTEM) image of a single nanorod. The interplanar spacing of the ordered stripes marked in Figure 3b is about 0.84 nm, which corresponds to the (001) lattice plane of Na_2_Ti_3_O_7_. The selected area electron diffraction (SAED) pattern further indicates that the nanorod is a monoclinic Na_2_Ti_3_O_7_. Na_2_Ti_3_O_7_ nanorods grow along the (010) direction. To further examine the distribution of the F elements, energy-dispersive X-ray spectrometry (EDX) mapping analysis was employed. The results demonstrate that Na, O, Ti, and F elements are uniformly distributed in Figure 3d, which indicates that F was doped into Na_2_Ti_3_O_7_.

### 3.2. Electrochemical Performance

**Figure 4:**
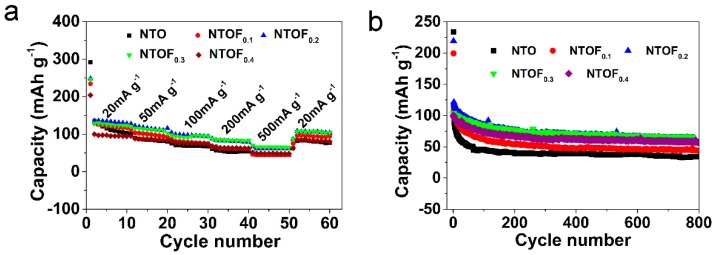
(**a**) The specific capacity performance of NTO and NTOF_x_ at different current densities and (**b**) the cycling property of NTO and NTOF_x_ at a current density of 100 mA g^−1^.

**Figure 5:**
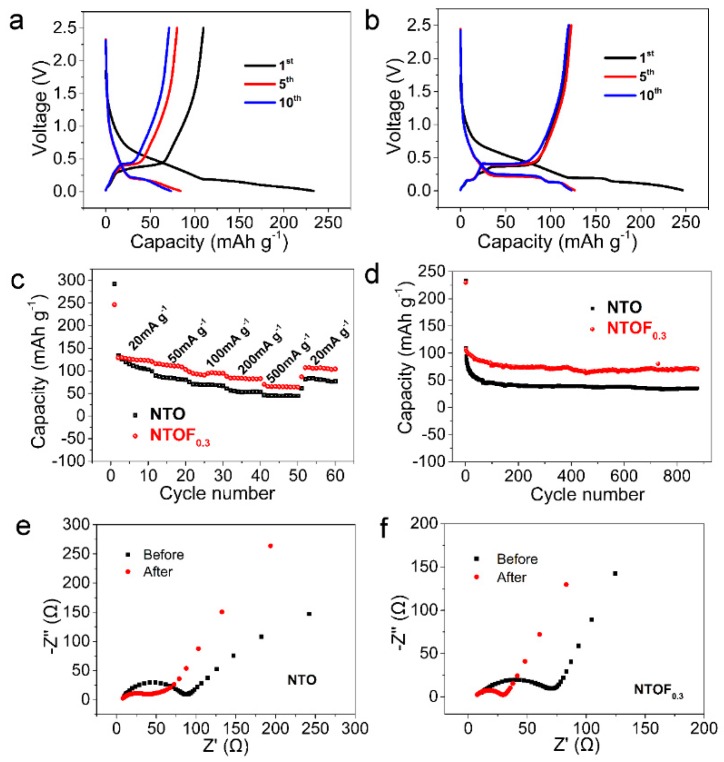
Electrochemical performances of NTO and NTOF_0.3_ electrodes. (**a**) Charge–discharge profiles of NTO; (**b**) charge–discharge profiles of NTOF_0.3_; (**c**) rate capability of NTO and NTOF_0.3_ electrodes; (**d**) cycling performance of NTO and NTOF_0.3_ electrodes; Nyquist plots of (**e**) NTO electrode and (**f**) NTOF_0.3_ electrode before and after the initial cycle at a current density of 100 mA g^−1^, tested under open circuit voltage conditions with a bias voltage of 5 mV.

In Figure 4a,b, the cell with an NTOF_0.3_ electrode exhibits the best electrochemical performance. Therefore, we mainly focused on the investigation of NTOF_0.3_. Figure 5a,b exhibits the charge and discharge curves of the NTO and NTOF_0.3_ electrodes at a current density of 20 mA g^−1^. The initial reversible discharge and charge capacities of NTO and NTOF_0.3_ are 233.4 and 109.7 mAh g^−1^; 246.3 and 120.6 mAh g^−1^, respectively. The initial coulombic efficiency is only 46.7% and 48.9% but it reaches almost 100% in subsequent cycles. The coulombic efficiency of the initial cycle may originate from the irreversible formation of a solid electrolyte interphase (SEI) film [29,30,31,32]. Due to the formation of the passivating layer on the surface and the reactive Ti–O that leads to electrolyte decomposition, the irreversible capacity loss in the titanium-based Na electrode is usually serious. However, after the initial cycle, the low irreversible capacity loss can be suppressed and thus high efficiency can be achieved when active Ti–O is passivated [33]. There is a sloping voltage plateau at approximately 0.46 V during the charge process, while a plateau is obtained at about 0.63 V during the discharge process. Furthermore, Figure 5c shows the rate capabilities of NTO and NTOF_0.3_ electrodes at current densities ranging from 20 mA g^−1^ to 500 mA g^−1^. It can be seen that the specific capacity of the NTOF_0.3_ electrode is much higher than that of the NTO electrode. Figure 5d displays the cycling performance of NTO and NTOF_0.3_ electrodes. Both electrodes can run stably for more than 800 cycles at 100 mA g^−1^. The discharge specific capacity of the NTOF_0.3_ electrode is about 30% higher than that of the NTO electrode. Figure 5e,f displays the Nyquist plots of NTO and NTOF_0.3_ electrodes, respectively. All the plots show a depressed semicircle in the high-frequency region and a sloping line in the low-frequency region. It is believed that the former corresponds to the charge–transfer resistance (*R*_CT_) while the latter corresponds to the Warburg diffusion process. It can be seen that the NTOF_0.3_ electrode exhibits smaller semicircles (72.1 Ω) at high and medium frequencies when compared to those of the NTO electrode (147.7 Ω), indicating that F-doping can decrease the charge–transfer resistance. Compared with the resistances of the electrodes before and after the initial cycle, both the resistances of NTO and NTOF_0.3_ after the first cycle are significantly decreased. This indicates that an activation process occurs during the cycling.

**Figure 6:**
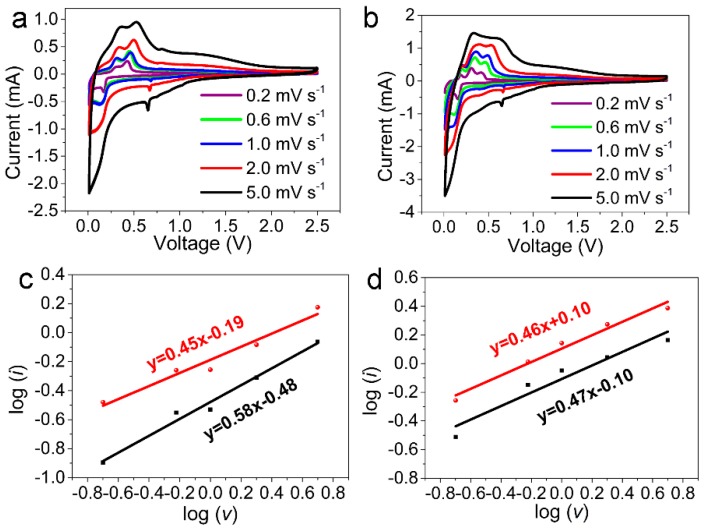
(**a**,**b**) Cyclic voltammetry (CV) curves of the NTO and NTOF_0.3_ electrodes at different scan rates; (**c**,**d**) the corresponding linear relationship between the logarithm of peak current and the logarithm of the scan rate (log *v*) for the NTO electrode and NTOF_0.3_ electrode.

To reveal why the NTOF_0.3_ electrode exhibits enhanced performance compared with the NTO electrode, the electrochemical kinetics of Na^+^ deintercalation and intercalation processes in Na_2_Ti_3_O_7_ were studied. Figure 6a,b shows the Cyclic voltammetry (CV) curves of the NTO and NTOF_0.3_ electrodes. A cathodic peak at 0.16 V and an anodic peak at 0.68 V versus Na/Na^+^ were observed, corresponding to typical Na^+^ insertion/extraction in the NTO lattice, which are in consistent with the charge and discharge curves. Significantly, a redox pair developed at 0.68 V and gradually augmented with the scanning rate. This redox may be ascribed to the storage of sodium with low-valence-state titanium atoms. These two redox peaks were assigned to the redox couple of Ti^4+^/Ti^3+^ over the discharge and charge processes. The reaction procedures can be depicted by Formula (1) [34]:

Na_2_Ti_3_O_7_ + x Na^+^ + x e^−^ → Na_2+x_Ti_3_O_7_ (0 < x < 3.5) (1)

The redox peaks of NTOF_0.3_ demonstrate its outstanding kinetic property. Figure 6c,d reveals the dependence of the logarithm of peak currents (log *i*) on the logarithm of the scan rates (log *v*). Furthermore, the reaction kinetics can be revealed by the formula of *i* = a*v*^b^, which can also be expressed as log *i* = b × log *v* + log a, where *i* is the peak current, a and b denote related parameters, and *ν* represents the sweep rate [35,36]. When the value of b is close to 0.5, a battery behavior dominates the process; when the b value approaches 1.0, it shows the behavior of a capacitor. In Figure 6c, the log *i* versus the log *v* shows a linear relationship. Thus, the b values of the two peaks at 0.25 V and 0.16 V can be calculated as 0.58 and 0.45, respectively, while the values of b of the two redox peaks for NTOF_0.3_ are 0.47 and 0.46, respectively. Therefore, these results indicate that a mixed process exists in the NTO and NTOF_0.3_ anodes, although a diffusion-controlled process via a capacitive Na^+^ storage mechanism should be more dominant.

According to Equation (2) [37]:

(2) $$I_{P}=2.69\times 10^{5}An^{\frac{3}{2}}C_{0}D^{\frac{1}{2}}v^{\frac{1}{2}}$$

where *I_p_* represents peak current (A), *A* is the electrode area (cm^2^), *n* is the number of electrons transferred, *C*_0_ is the concentration of Na^+^ ion (cm^2^ s^−1^), *D* is the diffusion coefficient of Na^+^ (mol cm^−3^), and *v* is the sweeping rate (V s^−1^) in CV. Thus, the calculated diffusion coefficients of Na ions in NTO and NTOF_0.3_ electrodes are 7.73 × 10^−9^ cm^2^ s^−1^ and 1.7 × 10^−8^ cm^2^ s^−1^, respectively. This result is consistent with the EIS results. These results indicate that F-doping is favourable for improving electrochemical performance.

## 4. Conclusions

In summary, F-doped Na_2_Ti_3_O_7_ nanorods were synthesized successfully by a solid-phase method. The effects of F-doping on the phase, morphology, and electrochemical performance of Na_2_Ti_3_O_7_ were investigated. F-doping does not change the crystal structure of NTO, although it has an effect on the morphology of the resultant product. The specific capacity of Na_2_Ti_3_O_7_ displays a 30% increase by F-doping due to the improved Na^+^ diffusion coefficient. F-doping can the charge–transfer resistance. The obtained material also shows a better rate capability and cycling performance for more than 800 cycles. F-doped Na_2_Ti_3_O_7_ nanorods are a promising anode for sodium-ion batteries (SIBs). This work provides a strategy for improving the electrochemical performance of the electrode materials.
